# Role of Prefrontal Cortex Anti- and Pro-inflammatory Cytokines in the Development of Abnormal Behaviors Induced by Disconnection of the Ventral Hippocampus in Neonate Rats

**DOI:** 10.3389/fnbeh.2018.00244

**Published:** 2018-10-22

**Authors:** Antoneta T. Joseph, Sanjeev K. Bhardwaj, Lalit K. Srivastava

**Affiliations:** Department of Psychiatry, Douglas Mental Health University Institute, McGill University, Montréal, QC, Canada

**Keywords:** Cytokines, TGF-β1, schizophrenia, animal model, ventral hippocampus, behavior

## Abstract

Neonatal disconnection of ventral hippocampus (VH) outputs in rats has been reported to lead to post-pubertal behavioral and synaptic changes of relevance to schizophrenia. Increased oxidative and inflammatory load in the prefrontal cortex (PFC) has been suggested to mediate some of the effects of neonatal VH lesion (NVHL). In this study, we hypothesized that developmental imbalance of anti- and pro-inflammatory factors within the PFC might affect synaptic development thus contributing to the adult NVHL-induced behavioral deficits. Ibotenic acid-induced excitotoxic NVHL was performed in postnatal day (PD) 7 male Sprague-Dawley rats and the mRNA levels of select pro- and anti-inflammatory cytokines were measured in the medial PFC (mPFC) at two developmental time points (PD15 and PD60). We observed a development-specific increase of pro-inflammatory cytokine, interleukin (IL)-1β mRNA at PD15, and an overall reduction in the expression and signaling of transforming growth factor beta 1 (TGF-β1), an anti-inflammatory cytokine, at both PD15 and PD60 in the NVHL animals. These cytokine changes were not seen in the somatosensory cortex (SSC) or tissue surrounding the lesion site. Daily administration of systemic recombinant TGF-β1 from PD7-14 prevented the appearance of hyperlocomotion, deficits in prepulse inhibition (PPI) of startle and social interaction (SI) in post-pubertal (PD60) NVHL rats. Neonatal supplementation of TGF-β1 was also able to attenuate dendritic spine loss in the layer 3 mPFC pyramidal neurons of NVHL animals. These results suggest that early damage of the VH has long-lasting inflammatory consequences in distant connected structures, and that TGF-β1 has potential to confer protection against the deleterious effects of developmental hippocampal damage.

## Introduction

Disrupted functional connectivity between the hippocampus and the prefrontal cortex (PFC), presumably of developmental origin, is considered a core deficit in schizophrenia and other neurodevelopmental psychiatric disorders (Godsil et al., 2013; Bähner and Meyer-Lindenberg, 2017). In the rodents, monosynaptic glutamatergic afferents from the CA1 and subiculum of the ventral hippocampus (VH) to the medial PFC (mPFC; Thierry et al., 2000), form a critical pathway mediating the communication between the two structures. This interaction between the VH and the mPFC begins early in life (first postnatal week) in the form of theta-gamma coupled oscillations (Brockmann et al., 2011). A number of studies have attempted to assess whether an early-life disruption of the VH output could be a mechanism underlying later development of abnormal behaviors and neural alterations observed in neurodevelopmental disorders such as schizophrenia. Indeed, several reports show that excitotoxic neonatal VH lesion (NVHL) in rat pups (PD7) leads to adult schizophrenia-related behavioral deficits in sensorimotor gating, social interaction (SI) and cognition (for reviews see Marcotte et al., 2001; Tseng et al., 2009).

While the VH is connected to several cortico-limbic regions, it is interesting that NVHL animals exhibit numerous cellular and synaptic changes in the PFC at adulthood. For example, reduced dendritic spine density in layer 3 and 5 pyramidal neurons in the mPFC, imbalance in excitatory and inhibitory synaptic transmission (Flores et al., 2005; Ryan et al., 2013), impaired synaptic plasticity (Bhardwaj et al., 2014) and alterations in parvalbumin (PV) interneurons (Behrens et al., 2007) have all been reported in adult animals following NVHL. However, local mechanisms within the PFC that drive aberrant neural development and behavior in NVHL model are poorly understood. Increase in inflammatory responses and oxidative stress were suggested to play a critical role in the abnormal maturation of PV neurons in NVHL rats (Cabungcal et al., 2014). Consistent with this idea, we recently reported a development- specific effect of the NVHL on mPFC microglial reactivity and expression of genes implicated in oxidative stress and synaptic pruning (Hui et al., under review). Given that a critical balance of pro- and anti-inflammatory cytokines is important for neural functions (Spulber et al., 2009; Yamato et al., 2014) and alterations in pro- and anti-inflammatory cytokines have been reported in the brain and cerebrospinal fluid (CSF) of schizophrenia patients (Trépanier et al., 2016; Wang and Miller, 2018), we hypothesized that alterations in this balance in NVHL animals could be involved in synaptic and behavioral changes.

In the brain, pro- and anti-inflammatory responses are critically regulated by microglia (Wang et al., 2015; Andreou et al., 2017). Increased expression of pro-inflammatory cytokines like interleukin (IL)-1β, IL-6 and tumor necrosis factor-α (TNF-α) play a detrimental role on the development of the brain (Deverman and Patterson, 2009; Wang et al., 2015). Overall the pro-inflammatory cytokines have a detrimental effect on dendritic spine development (Bitzer-Quintero and González-Burgos, 2012). IL-1β and IL-6 are immediate early proteins secreted by microglia, and have been found to induce oxidative stress and reduce dendritic length (Gilmore et al., 2004). Elevated levels of IL-6 within the brain leads to abnormality in the shape, length and distributing pattern of dendritic spines and also affects the excitatory/inhibitory synaptic neurotransmissions (Wei et al., 2012). TNF-α in conjunction with microglia is a key mediator affecting synaptic remodeling and thereby affecting long term potentiation (LTP) and long term depression (LTD; Goverman, 2009; Kondo et al., 2011). In contrast, anti-inflammatory cytokines belonging to the transforming growth factor beta (TGF-β) family as well as IL-10 and IL-1 receptor antagonist (IL-1RA) suppress inflammation and have been suggested to exert beneficial and neuroprotective actions (Dobolyi et al., 2012; Adzic et al., 2018). TGF-β1, in particular plays a role in neuronal development and synaptic plasticity as well as in neuroprotection and regulation of microglial physiology (Spittau and Krieglstein, 2012; Butovsky et al., 2014). For example, IL-1β induced microglial activation and its associated oxidative stress markers were found to be blocked by TGF-β1 (Basu et al., 2002), and upregulation of canonical TGF-β signaling pathway (via p-Smad-2/3) was found to contribute to quiescent microglia phenotype thereby reducing inflammatory overload with in central nervous system (CNS; Abutbul et al., 2012). Administration of exogenous TGF-β1 human recombinant protein was able to rescue the LTP and object recognition memory deficits which were caused by inhibiting TGF-β signaling. TGF-β1 has also been implicated in schizophrenia; a gene-set enrichment analysis of genome-wide association analysis data showed one of the pathways being related to TGF-β (Jia et al., 2010).

In this study, we assessed pro- and anti-inflammatory cytokines in the mPFC of NVHL rats and asked whether neonatal administration of recombinant TGF-β1 could rescue the behavioral and cellular deficits observed in the lesioned rats at post-pubertal ages.

## Materials and Methods

### Neonatal Ventral Hippocampal Lesion (NVHL) and TGF-β1 Treatment

This study was carried out in accordance with the guidelines of the Canadian Council of Animal Care. The protocol was approved by the McGill University Animal Care Committee. Twenty pregnant female Sprague-Dawley rats at 15–18 days of gestation were obtained from Charles River Laboratories (QC, Canada). Rat dams were housed individually on a 12 h light-dark cycle with *ad libitum* food and water in a temperature and humidity-regulated room, where they were allowed to give birth. On PD7, male pups were weighed (15–17 g) and were randomly selected for neonatal ventral hippocampal lesion (NVHL) or sham surgery according to our previously described procedures (Flores et al., 1996; Ryan et al., 2013). Briefly, pups were anesthetized by hypothermia (by covering in crushed ice for 18–20 min) and secured on a modified platform fixed to Kopf stereotaxic apparatus. The “lesion” group received bilateral infusion of 0.3 μl ibotenic acid (Catalog No. 0285; Tocris; 10 μg/μl in 0.1 M phosphate-buffered saline (PBS) over a period of 2 min using a 30-gauge needle connected to an infusion pump, while the “sham” group received the same volume of PBS into the VH (coordinates: AP −3.0 mm from Bregma, ML ±3.5 mm from midline, and DV −5.0 mm from dura). Following this, the skin was sutured using vetbond tissue glue and the pup’s ears were tagged. After surgery, pups were placed on a heating pad until full recovery and returned to their respective mothers.

Separate cohorts of sham and lesioned animals were used for measurement of cytokines level (IL-1β, IL-6, TNF-α, IL-10, TGF-β1 and IL-1RA) and TGF-β1 signaling proteins at neonatal (PD15) and young adult (PD60) ages. Another cohort of sham and lesioned animals were randomly divided into two groups, one receiving recombinant TGF-β1 (Catalog No. T7039; Sigma (200 ng/kg; i.p.)) or equivalent volume of saline. TGF-β1 was administered 2 h before NVHL surgery and continued for next 7 days (from PD8-PD14). At PD21 the animals were weaned and housed in pairs. At adulthood (PD60), all four groups of animals (Sham-Saline, Sham-TGF-β1, Lesion-Saline and Lesion-TGF-β1) were subjected to behavioral tests followed by cytokine measurements.

### RNA Extraction and Cytokine Gene Expression Using QRT PCR

Animals were sacrificed by decapitation and their brains were removed and sliced into 1 mm thick slices. From one cohort of animals (Sham and lesion) medial PFC (infralimbic and prelimbic cortices) and two control brain regions (Somatosensory cortex (SSC) and the region around VH) were micropunched and the tissues of both hemispheres from each animal were pooled and stored at −80°C until use. Another cohort of animals which were neonatally administered with saline or recombinant TGF-β1 was sacrificed after the behavioral tests. Their brains were extracted and only mPFC micropunched and stored at −80°C until further use. RNA extraction was performed using Trizol reagent (Catalog No. 15596026; ThermoFisher Scientific) with the Purelink RNA minikit (Catalog No.12183018A; ThermoFisher Scientific). Yield and the quality of the RNA was determined using Nanodrop and agarose gel electrophoresis. Two microgram of RNA was used for the cDNA synthesis using the high capacity cDNA reverse transcription kit (Catalog No. 4368814; Applied Biosystem).

Primers for IL-1β (F-CACCTCTCAAGCAGAGCACAGR-GGGTTCCATGGTGAAGTCAAC), IL-6 (F-GCCCTTCAGGAACAGCTATGA R-TGTCAACAACATCAGTCCCAAGA), TNF-α (F-CCAGGAGAAAGTCAGCCTCCT R-TCATACCAGGGCTTGAGCTCA), TGF-β1 (F-TGGCGTTACCTTGGTAACC R-GGTGTTGAGCCCTTTCCAG), IL-10 (F-AAAGCAAGGCAGTGGAGCAG R-TCAAACTCATTCATGGCCTTGT), IL-1RA (F-GAGACAGGCCCTACCACCAG R-CGGGATGATCAGCCTCTAGTGT) and a housekeeping gene GAPDH (F-AGCCCAGAACATCATCCCTG R-CACCACCTTCTTGATGTCATC) were designed using the NCBI DNA sequence and the primer express software. QRT PCR was performed using SYBR green PCR master mix (Catalog No. A6001; Promega) according to the manufacturer’s protocol. The following cycling conditions were used in Applied Biosystem Real time PCR 7,500 machine. Initial denaturation at 95°C for 10 min, followed by 40 cycles with denaturation at 95°C for 15 s, annealing at 60°C for 1 min and elongation at 72°C for 1 min. A standard and a melting curve for all the genes were obtained to check the efficiency of the primers. 2^−ΔΔCT^ method was used to calculate the fold changes.

### Immunoblotting for TGF-β1 Signaling Proteins

Sham and NVHL animals at PD60 were sacrificed by decapitation and the brains were sliced into 1 mm thick slices. The area corresponding to the prelimbic and infralimbic mPFC was rapidly dissected and homogenized in Tris EDTA buffer (containing PMSF (0.2 mM), leupeptin (1 mM), pepstatin (1 mM), sodium orthovanadate (1 mM) and SDS (0.1%)). Western blotting was done as described previously (Bhardwaj et al., 2014). A 15 μg protein was electrophoresed on 4%–20% Tris-glycine gels and blotted to nitrocellulose membranes. The blots were incubated with 1:2,500 dilution of rabbit polyclonal antibody against phospho-Smad-2/3 (Catalog No. 8828S; Cell Signaling) or total-Smad-4 (Catalog No. 49515S; Cell Signaling) followed by anti-rabbit IgG: peroxidase-linked second antibody (Catalog No. 7074; Cell Signaling, 1:2,500). The blots were developed using chemiluminescence detection system (Catalog No. NEL103E001EA; Perkin-Elmer) and exposed to X-ray film. After phospho-Smad-2/3 probing, the blot was stripped and probed for total-Smad-2/3 (Catalog No. 5678S; Cell Signaling) followed by restriping and reprobing for α-tubulin antibody (Catalog No. T5168; Sigma, 1:5,000). Relative optical densities (ROD) of bands were analyzed on image analysis system (MCID-4, Imaging Research).

In order to verify whether exogenously administered TGF-β1 at neonatal ages directly modifies TGF-β signaling in the brain, another set of control animals were injected with either recombinant TGF-β1 (5 μg) or saline intraperitoneally at PD7 and the animals were sacrificed 2 h later. The mPFC was extracted and homogenized to measure the level of Smad-2/3 phosphorylation by Western blotting as described above.

### Behavioral Testing

Behavioral testing was performed at PD60; the same sets of animals were used for all behavioral tests except a few animals which had to be excluded due to ill-health. The tests were performed during the light phase of the normal 12 h light-dark cycle (lights on at 8:00 and off at 20:00) starting with the least stressful, in the order presented below. Tests were separated by at least 72 h.

#### Spontaneous Locomotor Activity

The spontaneous locomotor activity was assessed as described previously (Bhardwaj et al., 2012), using acrylic activity chambers (AccuScan Instruments Inc., Columbus, OH, USA; L × W × H = 40 × 40 × 30 cm) in a dimly lit room. The chambers area was equipped with infrared sensors. Animals were brought from their home environment to the testing room and immediately placed in the activity boxes where their activity was monitored during the next 60 min. Data was collected using the Versamax Software. The total horizontal activity in the whole session (60 min) was used to analyze the locomotor behavior.

#### Social Interaction (SI)

The three-chamber method as described previously (McKibben et al., 2014) was used to measure the sociability of the rodents with a conspecific as well as social discrimination memory of familiar vs. novel conspecific rat. Two wire mesh cages (20 cm × 20 cm × 15 cm) were placed in two chambers on the right and left of the central chamber and the central chamber was devoid of any objects. This method involved three steps; one, habituation where the test animals were placed in the central chamber and allowed to explore all the chambers for 10 min. After this the animals were placed back in their home cage. For the testing procedure, a stranger (S1) or an unfamiliar rat (same strain, sex and age) was placed in any one of the wire mesh cage and the test animals were reintroduced in the central chamber and allowed to explore all the chambers for 5 min. The interaction between the test animal and the stranger (S1) as compared to the empty wire mesh cage was a measure of sociability. For the measure of social novelty preference or social memory, after the first 5 min of interaction, the test animal was placed back in the home cage for 5 min (Retention interval) during which another novel stranger (S2) or unfamiliar rat was introduced in the second wire mesh cage.

After the retention interval, the test animal was reintroduced into the central chamber and was allowed to explore all the chambers for 5 min. The animal behavior was recorded and the interaction with S1 and S2 rat was measured and scored as sociability and social memory respectively. The interaction between the two rats was measured by nose contacts, sniffing within a distance of 1 cm. The videos were scored in a “blinded” manner for the total time spent interacting. This time measured was referred to as exploration time. Sociability and social memory analysis were carried out by calculating the exploration ratio. The sociability exploration ratio was calculated with the following equation: time spent with stranger 1 (S1)/Total time of interaction (S1 + empty mesh cage) * 100. The following equation was used to measure social memory: time spent with the novel animal (S2)/Total time of interaction (S1+ S2) * 100. Total time of interaction is the time spent by the test animal in interacting with the familiar animal (S1) and the novel animal (S2).

#### Prepulse Inhibition (PPI)

As described previously (Bhardwaj et al., 2012), we used a commercially available system (SR-LAB; San Diego Instruments, San Diego, CA, USA) equipped with a cylindrical Plexiglas animal enclosure and a small electric fan, which generated a 70 dB background noise and provided ventilation to measure prepulse inhibition (PPI) in sound-attenuating chambers. Sound pressure levels (dB(A) weighting) were measured at the position of the rat’s ears. Broadband noise pulses were presented via a speaker positioned directly above the animal. An accelerometer affixed to the animal enclosure frame was used to detect and transduce motion resulting from the animal’s response. Noise pulse parameters were controlled using SR-LAB software, which also recorded responses. Animals were acclimated to the enclosure for 5 min before being tested during 37 discrete trials. On the first two trials, the magnitude of the startle response to a 120-dB white noise pulse was measured. These first two startling pulses were presented to habituate the animals to the testing procedure and thus were omitted from the data analysis; all subsequent trials were included in analysis. On the subsequent 35 trials, the startle pulse was either presented alone or 100 ms after the presentation of 30 ms prepulse. Acoustic startle response (ASR) to the pulse was measured following trials with prepulse intensities of 6, 9, 12 and 15 dB above background noise. Prepulses were varied randomly between trials, and each prepulse was presented five times; animals were randomly presented with the startle pulse alone during the other 10 trials. The average inter-trial interval was 15 s (range, 5–30 s). Startle responses were determined automatically by the SR-LAB analysis suite. Startle magnitude was calculated as the average of the startle responses to the pulse-alone trials. PPI was calculated according the formula: %PPI = 100 − (startle response for prepulse + pulse trials/startle response for pulse alone trials) × 100%.

### Golgi Cox Staining

After the behavioral testing, few animals from each group were used for Golgi-Cox staining to assess the morphological changes including dendritic complexity and spine density in mPFC following NVHL and TGF-β1 administration. 48–72 h after the last behavior testing, animals were rapidly decapitated and the brains were extracted and washed with chilled Milli-Q water. Golgi impregnation was performed according to the specifications of the FD Rapid GolgiStain kit (Catalog No. PK401FD; NeuroTechnologies, INC) with the following optimizations: whole brains were treated for silver impregnation for 12 days, cryoprotected with 30% sucrose solution for 72 h, and sectioned at 200 μm in a vibratome in a 6% sucrose solution. Brain sections were mounted on gelatin-coated slides, lightly pressed and kept in moist container until developed, clarified, and then cover slipped using Permount following FD Rapid GolgiStain kit guidelines. The layer III pyramidal neurons (10 neurons per animal) from the mPFC were traced and a three-dimensional reconstruction of the neurons was carried out using the Neurolucida software (Leica microscope). Mean values of total basilar dendritic length and spine density were calculated. For dendrite arborization pattern, Sholl analysis (number of intersections per each radius 5 μm) was employed as described previously (Baharnoori et al., 2009).

### Histological Examination

Following sacrifice and brain extraction, 35 micron coronal sections at the level of the VH were mounted on pre-coated microscope slides and stained with cresyl violet staining solution (0.5%) for verification of the lesion. Only animals with bilateral lesions confined to the VH with no significant damage to the dorsal part or adjacent thalamic nuclei were included in data analyses.

### Data Analysis

All reported values are mean ± standard error of the mean (SEM). All data except dendritic morphology were analyzed using Prism (GraphPad, Version 6). Student *t*-test (two tailed) was used to determine gene and protein expression changes in sham and lesioned animals. Two-way ANOVAs followed by tukey’s *post hoc* tests were used to determine interactions between lesion status and TGF-β1 treatment. Three-way ANOVA was used to determine interaction between lesion, TGF-β1 treatment, timeline of horizontal activity and prepulse intensities. General Linear Model (repeated measure) in SPSS was used to analyze the interaction of lesion and TGF-β1 treatment on dendritic morphology (Sholl analysis). Greenhouse-Guiser corrected F and *df* are reported for the general linear model analysis. For all analysis *p* < 0.05 was considered statistically significant.

## Results

### Lesion Verification

**Figure 1:**
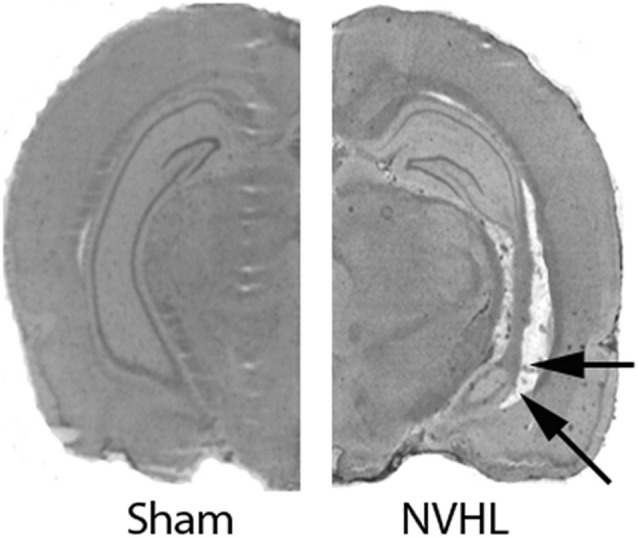
Verification of neonatal ventral hippocampus lesion (NVHL). Cresyl violet stained representative images showing an intact VH in sham animals (left). NVHL brain (right) shows cell loss, cavity and disorganization in the VH (as shown by arrows).

As reported earlier from our lab (Bhardwaj et al., 2012, 2014), NVHL animals showed bilateral neuronal loss, retraction, and cavitation’s in the ventral half of the hippocampus including the CA1 (Figure 1) of NVHL animals, but not of sham-operated animals. The lesion spared the dorsal hippocampus and the adjacent nuclei (i.e., amygdala and thalamus).

### An Imbalance in the Expression of Pro- and Anti-inflammatory Cytokines in PFC of NVHL Rat

**Figure 2:**
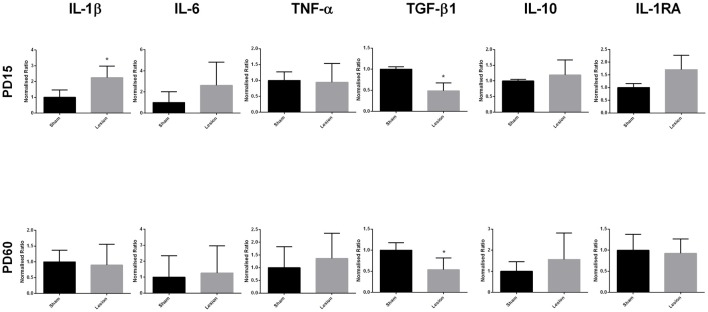
Levels of expression of pro- and anti-inflammatory cytokines from sham and NVHL animals from medial prefrontal cortex (mPFC) at PD15 and PD60. Quantification of Q-RTPCR data (normalized ratio of gene of interest over housekeeping gene); genes of interest quantified include interleukin-1β (IL-1β), interleukin-6 (IL-6), tumor necrosis factor-α (TNF-α), transforming growth factor-β1 (TGF-β1), interleukin-10 (IL-10) and interleukin-1RA (IL-1RA) at two developmental time points PD15 and PD60. Expression of IL-1β was found to be significantly increased in the NVHL groups as compared to the sham group at PD15 (*p = 0.0019). Decreased expression of TGF-β1 was observed in the NVHL group as compared to sham group both at PD15 (*p = 0.0291) and PD60 (*p = 0.0064). No significant change in the expression of IL-6, TNF-α, IL-10, IL-1RA was observed between sham and lesion group at PD15 and PD60 (*n* = 4–8 per group).

Analysis of the mRNA expression data at PD15 (Figure 2) showed a significant increase in the expression of pro-inflammatory marker IL-1β in mPFC of the NVHL rats (*t* = 4.839, *df* = 7, *p* = 0.0019); however, the expression of the other pro-inflammatory cytokine, IL-6 (*t* = 1.350, *df* = 3, *p* = 0.2697) and TNF-α (*t* = 2.672, *df* = 3, *p* = 0.0756) were not affected. On the contrary, we found a significant reduction in the expression of anti-inflammatory marker TGF-β1 (*t* = 5.735, *df* = 2, *p* = 0.0291) in lesioned animals. The expression of other anti-inflammatory markers, IL-10 (*t* = 0.6837, *df* = 2, *p* = 0.5647) and IL-1RA (*t* = 1.828, *df* = 2, *p* = 0.2090) remained comparable between sham and lesioned animals. At young adulthood (PD60), data analysis did not reveal any significant effect of lesion on the expression of IL-1β (*t* = 0.2532, *df* = 4, *p* = 0.8126), IL-6 (*t* = 0.3160, *df* = 4, *p* = 0.7678), TNF-α (*t* = 0.6061 *df* = 4 *p* = 0.5771), IL-10 (*t* = 1.295 *df* = 4, *p* = 0.2652) and IL-1RA (*t* = 0.3093, *df* = 4, *p* = 0.7725), whereas a significant reduction in TGF-β1 expression (*t* = 5.222, *df* = 4, *p* = 0.0064) in NVHL rats still persisted.

**Figure 3:**
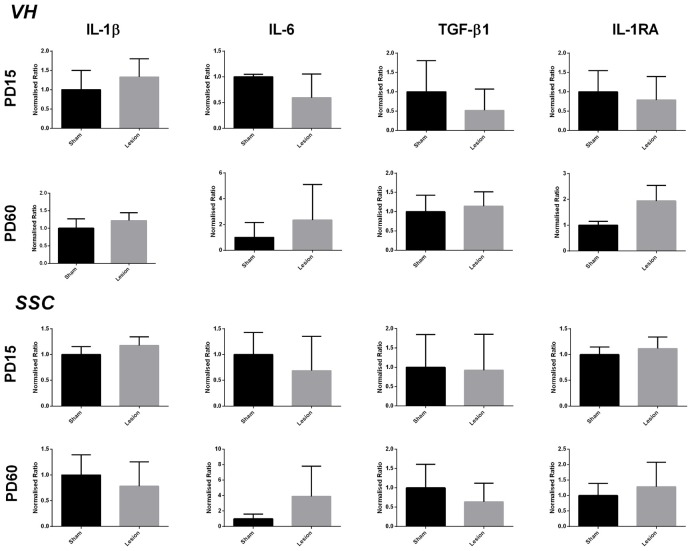
Levels of expression of pro- and anti-inflammatory factors from sham and NVHL animals within VH and somatosensory cortex (SSC) at PD15 and PD60. Quantification of Q-RTPCR data (normalized ratio of gene of interest over housekeeping gene); genes of interest quantified include IL-1β, IL-6, TGF-β1 and IL-1RA at two developmental time points PD15 and PD60. No significant change was observed in the expression of above mentioned genes between sham and lesion at PD15 and PD60 (*n* = 3–4 per group).

The data analysis from the area around VH showed no significant difference between the sham and lesion animals in the expression of IL-1β (*t* = 1.948, *df* = 3, *p* = 0.1466), IL-6 (*t* = 1.186, *df* = 3, *p* = 0.3212), TGF-β1 (*t* = 1.580, *df* = 3, *p* = 0.2122), IL-1RA (*t* = 1.024, *df* = 3, *p* = 0.3813) at PD15 or at PD60 (IL-1β (*t* = 0.9057, *df* = 3, *p* = 0.4318); IL-6 (*t* = 0.6960, *df* = 3, *p* = 0.5365); TGF-β1 (*t* = 0.5643, *df* = 3, *p* = 0.6120); IL-1RA (*t* = 2.742, *df* = 3, *p* = 0.0712)). The data analysis from PD15 sham and lesioned animals from SSC brain region did not reveal any significant effect on the expression of IL-1β (*t* = 1.332, *df* = 3, *p* = 0.2749), IL-6 (*t* = 1.015, *df* = 2, *p* = 0.4169), TGF-β1 (*t* = 0.3362, *df* = 3, *p* = 0.7589) and IL-1RA (*t* = 1.403, *df* = 3, *p* = 0.2552). Similar to the neonatal (PD15) group, the data analysis from postpubertal (PD60) animal’s SSC also did not reveal any significance between sham and lesioned animals; (IL-1β (*t* = 2.005, *df* = 3, *p* = 0.1386), IL-6 (*t* = 0.9785, *df* = 3, *p* = 0.4000), TGF-β1 (*t* = 1.282, *df* = 3, *p* = 0.2900) IL-1RA (*t* = 0.9438, *df* = 3, *p* = 0.4149)). The results are shown in Figure 3.

### Decrease in TGF-β Signaling in the mPFC of NVHL Animals

**Figure 4:**
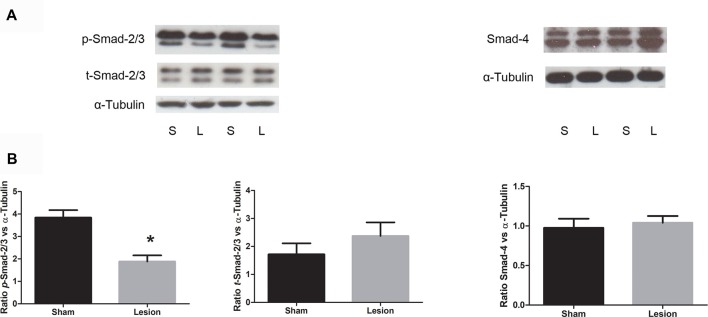
Expression of TGF-β signaling protein from sham and NVHL animals from medial PFC at PD60. **(A)** Representative Western blots showing the expression of phospho (p)-Smad2/3, (total) t-Smad-2/3, total-Smad-4 and α-tubulin in samples prepared from medial PFC tissue of sham (S) and NVHL (L) rats. The blot was developed for p-Smad-2/3, stripped and probed for t-Smad-2/3 and restriped and reprobed for α-tubulin antibody. **(B)** Quantitation of Western blotting data (relative optical densities (ROD) ratio of p-Smad-2/3, t-Smad-2/3 and Smad-4 over α-tubulin). A significant decrease in p-Smad-2/3 was observed in NVHL rats (*p = 0.003). No significant change in the normalized levels of t-Smad-2/3 and Smad-4 was observed between sham and lesion group (*n* = 5–6 per group).

In order to further investigate whether the decrease in mPFC TGF-β1 mRNA expression has functional significance, we assessed TGF-β canonical signaling components, phospho-Smad (*p-*Smad-2/3), total-Smad (t-Smad-2/3) and Smad-4 from mPFC, using Western blotting in PD60 sham and lesioned rats. Consistent with mRNA levels, data analysis revealed a significant decrease in the normalized phosphorylated Smad-2/3 protein levels in NVHL rats compared to sham rats (*t* = 4.53, *df* = 8, *p* = 0.002). No significant change in the levels of t-Smad-2/3 (*t* = 1.04, *df* = 8, *p* = 0.32) or Smad-4 (*t* = 0.4509, *df* = 10, *p* = 0.6617) was observed. The results are shown in Figure 4.

### Systemic Neonatal TGF-β1 Administration Modulates TGF-β Signaling in Brain

**Figure 5:**
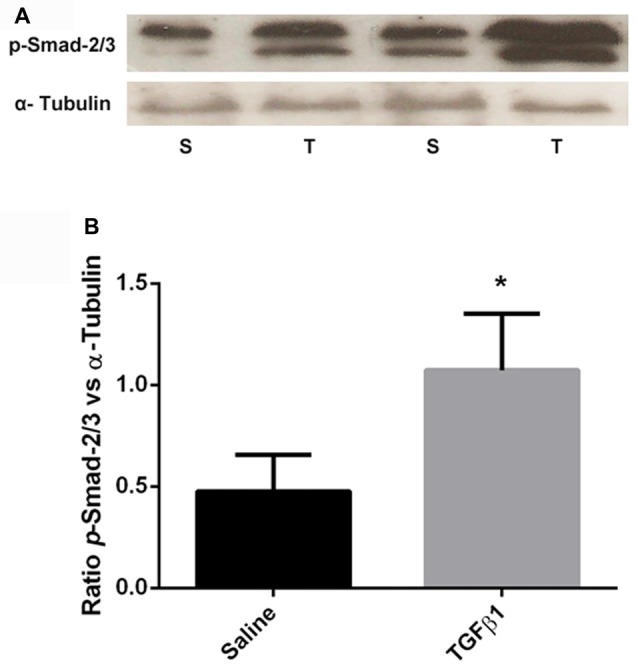
A single acute systemic injection of TGF-β1 regulates its brain intracellular signaling. Panel **(A)** shows the representative Western blot images showing the increased expression of p-Smad-2/3 in TGF-β1 (T) injected group compared to saline (S) injected group. **(B)** The quantification of the Western blotting data (ROD ratio of p-Smad2/3 over α-tubulin). A significant increase in p-Smad-2/3 was observed in TGF-β1 injected group as compared to saline injected group (*p = 0.011; *n* = 4 per group).

In order to assess if exogenous TGF-β1 treatment at neonatal age affects TGF-β signaling in the brain, we gave single high dose (5 μg) of TGF-β1 or saline intraperitoneally at PD7 to control rat pups. Two hours after the injection, pups were sacrificed, brains were removed and their mPFC was isolated and processed for phospho-Smad-2/3 Western blotting. Student’s *t*-test analyses of protein levels of phospho-Smad-2/3 ROD data (normalized to α-tubulin) showed a significant increase (*t* = 3.607, *df* = 6, *p* = 0.011) in the protein levels of phospho-Smad-2/3 following TGF-β1 administration compared to saline injections (Figure 5B).

### The Effect of Neonatal Recombinant TGF-β1 Administration on the Behavior of NVHL Animals at PD60

#### Locomotor Activity

**Figure 6:**
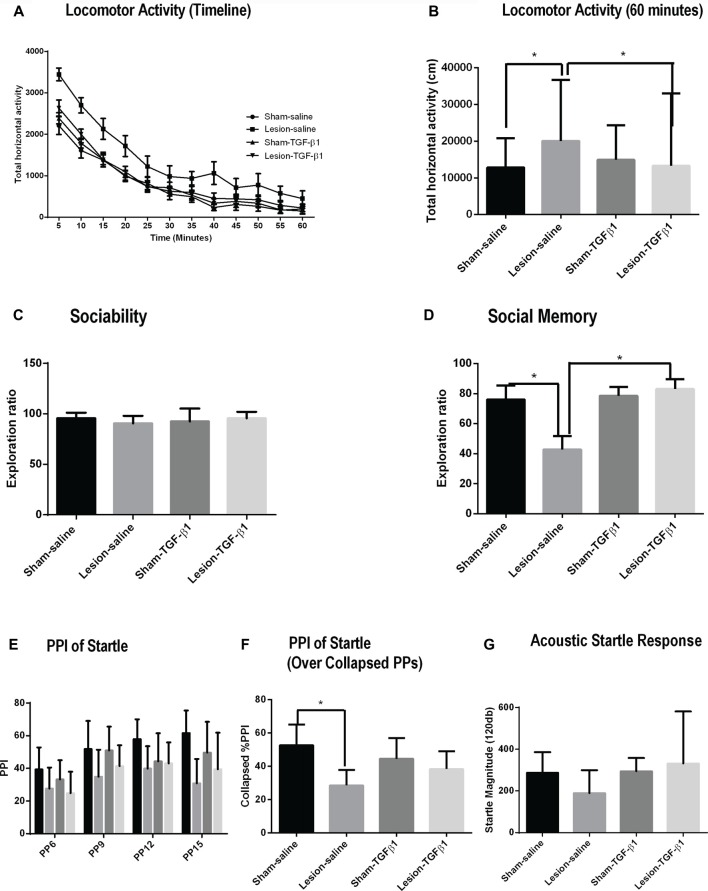
Effect of neonatal TGF-β1 administration on sham and NVHL animals at PD60. **(A,B)** Spontaneous locomotor activity. **(A)** Time course of locomotor activity as assessed by the horizontal activity in 5 min bin. **(B)** Total horizontal activity during the whole 60-min session. Two-way ANOVA showed a significant lesion × TGF-β1 treatment interactions on the horizontal activity (*p = 0.018). **(C,D)** Sociability and social memory **(C)** Indicates similar preferences between sham and NVHL animals for S1 animals. **(D)** In social memory test, significant lesion × TGF-β1 interactions was observed (*p = 0.017). **(E–G)** Prepulse inhibition (PPI) of acoustic startle response (ASR). **(E)** PPI, as a function of prepulse intensities (PP;6–15). **(F)** PPI collapsed across all PPs. A significant 2-way (lesion × TGF-β1) interactions was observed (*p = 0.026). **(G)** Baseline ASR was not significantly different between groups (*n* = 7–18 per group).

A three-way repeated measure ANOVA on horizontal activity of all the animals over time showed no significant three-way interaction on lesion × TGF-β1 treatment × timeline of horizontal activity (*F*_(1,605)_ = 0.53, *p* = 0.883; Figure 6A). Further analysis was performed on the total horizontal activity for the whole session (Figure 6B), which showed a sustained increased activity in saline treated lesioned animals. More specifically, two-way ANOVA of the data for the whole session (i.e., 60 min) revealed a significant main effect of lesion (*F*_(1,55)_ = 9.96, *p* = 0.002), TGF-β1 treatment (*F*_(1,55)_ = 7.59, *p* = 0.008) and lesion × TGF-β1 interaction (*F*_(1,55)_ = 5.85, *p* = 0.018). As reported previously in this model, *post hoc* tukey’s test revealed hyper-locomotion in saline-treated NVHL animals compared to saline-treated sham animals (*p* = 0.0021). Further *post hoc* analysis showed that while neonatal TGF-β1 treatment had no significant effect in sham animals, it led to a significant attenuation of locomotor activity in NVHL animals as compared to lesion-saline animals (*p* = 0.0031).

#### Social Interaction (SI)

Two-way ANOVA for sociability did not reveal any significant main effect of lesion (*F*_(1,37)_ = 0.1343, *p* = 0.7161), treatment (*F*_(1,37)_ = 0.09869, *p* = 0.7552) and lesion × TGF-β1 treatment interaction (*F*_(1,37)_ = 2.218, *p* = 0.1448); indicating that all groups of animals spend about the same amount of time interacting with S1 animal (Figure 6C). Social memory measured from PD60 sham and NVHL animals showed a significant main effect of TGF-β1 treatment (*F*_(1,37)_ = 7.782, *p* = 0.0083) and lesion × TGF-β1 treatment interaction (*F*_(1,37)_ = 6.166, *p* = 0.0177) but no significant main effect of lesion (*F*_(1,37)_ = 3.565, *p* = 0.0669). *Post hoc* tukey’s test showed a significant reduction in social memory in the saline treated NVHL animals compared to the Sham-saline group (*p* = 0.0249). This reduction in social memory was significantly reversed after the neonatal administration of TGF-β1 in lesioned animals (*p* = 0.0040) compared to lesion-saline group (Figure 6D).

#### Prepulse Inhibition (PPI) of Acoustic Startle Response (ASR)

A three-way repeated measure ANOVA on %PPI across all PP intensities (PP6-15) did not show any significant lesion × TGF-β1 treatment × PP intensities interaction (*F*_(1,96)_ = 1.10, *p* = 0.35458; Figure 6E). %PPI data with all PPs collapsed were then analyzed using a two-way ANOVA (Figure 6F). Analysis from %PPI on collapsed prepulse intensities showed a significant main effect of lesion (*F*_(1,32)_ = 15.35, *p* = 0.0004), and lesion × TGF-β1 treatment interaction (*F*_(1,32)_ = 5.29, *p* = 0.027) but no TGF-β1 treatment (*F*_(1,32)_ = 0.047, *p* = 0.829). Tukey’s *post hoc* tests showed that there is a significant deficit in %PPI in lesion-saline animals compared to sham-saline animals (*p* = 0.001). Further *post hoc* test revealed that neonatal TGF-β1 treatment had no effect on PPI deficit on any group of animals (Figure 6F). Two way ANOVA on ASR data did not reveal any significant main effect of either lesion (*F*_(1,31)_ = 0.2166, *p* = 0.6449), treatment (*F*_(1,31)_ = 1.325, *p* = 0.2585) or lesion × TGF-β1 treatment interaction (*F*_(1,31)_ = 1.085, *p* = 0.30560; Figure 6G).

### Dendritic Complexity and Spine Density

**Figure 7:**
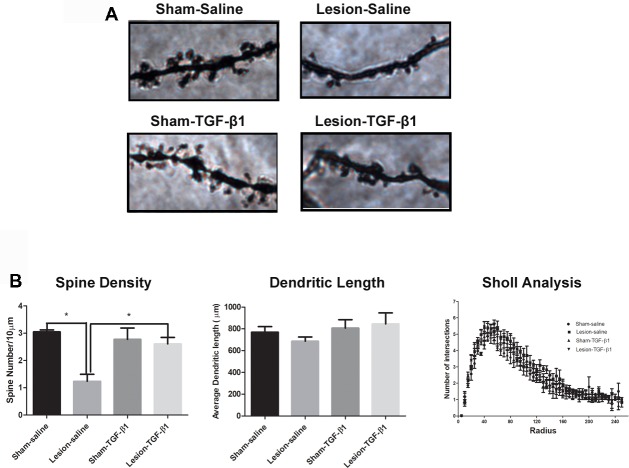
Effect of neonatal TGF-β1 administration on dendritic complexity and spine density in sham and NVHL animals within medial PFC at PD60. **(A)** Photomicrograph showing representative golgi-cox impregnated basilar dendrite from pyramidal neuron of layer III medial PFC at PD60 in sham-saline, lesion-saline, sham- TGF-β1 and lesion- TGF-β1 groups. **(B)** Two-way ANOVA showed a significant lesion × TGF-β1 treatment interaction on dendritic spine density (number of spines per 10 μm; *p = 0.02). Total dendritic length (μm) did not change significantly as a result of lesion × TGF-β1 treatment interaction among the four groups (*p = 0.41). Data analysis from dendritic complexity as measured by sholl analysis (Number of dendritic intersections per each sholl radius 5 μm) did not reveal any significant lesion × TGF-β1 treatment × dendritic intersections interaction between the four groups (*n* = 3–4 per group).

We further examined the dendritic complexity and spine density of adult sham and NVHL animals following the neonatal TGF-β1 treatment. Figure 7A shows the representative photomicrographs of Golgi-stained mPFC pyramidal neurons of saline as well as TGF-β1 administered sham and lesioned animals at adulthood. Two-way analysis on dendritic spine density (number of spines per 10 μm) revealed a significant main effect of lesion (*F*_(1,10)_ = 10.28, *p* = 0.009) and lesion × TGF-β1 treatment interactions (*F*_(1,10)_ = 7.07, *p* = 0.02) but no main effect of TGF-β1 treatment (*F*_(1,10)_ = 3.108, *p* = 0.1084). As reported previously, *post hoc* tukey’s test revealed, that spine density was significantly reduced from layer III pyramidal neurons of saline treated NVHL animals compared to sham-saline animals (*p* = 0.002). Further, data analysis revealed that while neonatal TGF-β1 treatment had no effect in sham animals, it rescued the spine density loss in lesion-TGF-β1 animals compared to lesion-saline group (*p* = 0.010). Two-way ANOVA on the dendritic length (μm) between sham and lesion animals however, did not show significant main effect of lesion (*F*_(1,10)_ = 0.10, *p* = 0.75), TGF-β1 treatment (*F*_(1,10)_ = 1.9, *p* = 0.19) or lesion × treatment interaction (*F*_(1,10)_ = 0.73, *p* = 0.41). Similarly, the analysis on the dendritic complexity (Sholl analysis), did not show any significant main effect of either lesion (*F*_(5.8,58)_ = 1.18, *p* = 0.32), treatment (*F*_(5.8,58)_ = 1.46, *p* = 0.21), or lesion × treatment × intersections interaction (*F*_(5.8,58)_ = 0.65, *p* = 0.68). Whereas the main effect of dendrite intersections within different group was found to be significant (*F*_(5.8,58)_ = 128.87, *p* = 0.00). Results are shown in Figure 7B.

### Effects of Neonatal TGF-β1 Administration on mPFC IL-1 β Levels

**Figure 8:**
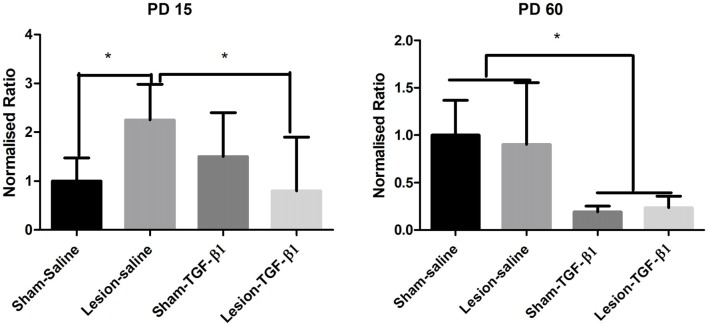
Levels of expression of IL-1β from sham and NVHL animals from medial PFC at PD15 and PD60 following neonatal TGF-β1 administration. Quantification of Q-RTPCR data (normalized ratio of IL-1β gene over housekeeping gene) expression at two developmental time points PD15 and PD60. A two-way ANOVA revealed a significant lesion × TGF-B1 interaction from PD15 group of animals (*p = 0.007). *Post hoc* test showed that IL-1β expression was significantly increased in the lesion-saline group as compared to sham-saline group (*p = 0.0169) at PD15. A significant reduction of IL-1β was observed in the lesion-TGF-β1 group as compared to lesion-saline group (*p = 0.0244). From PD60 group of animals only a significant main effect of TGF-β1 treatment was observed on IL-1β expression (*p = 0.001) (*n* = 4–8 per group).

Two-way ANOVA from PD15 group of animals following TGF-β1 administration did not reveal any main effect of either lesion (*F*_(1,20)_ = 0.7030, *p* = 0.4117) or TGF-β1 treatment (*F*_(1,20)_ = 2.122, *p* = 0.1607) but a significant lesion × treatment interaction (*F*_(1,20)_ = 8.910, *p* = 0.0073) was observed in IL-1β mRNA expression. Tukey’s *post hoc* test showed a significantly increased IL-1β mRNA expression in lesion-saline group compared to sham-saline animals (*p* = 0.0169). Further analysis showed that while TGF-β1 administration had no effect on sham animals, it led to significantly attenuated IL-1β mRNA expression in lesion-TGF-β1 administered group compared to saline administered—lesioned animals (*p* = 0.0244). Two-way analysis on PD60 group of sham and lesioned animals revealed only a significant main effect of TGF-β1 treatment on IL-1β mRNA expression (*F*_(1,15)_ = 16.56, *p* = 0.0010). No significant main effect of lesion (*F*_(1,15)_ = 0.02207, *p* = 0.8839) or lesion × TGF-β1 treatment interaction (*F*_(1,15)_ = 0.1568, *p* = 0.6977) was observed (Figure 8).

## Discussion

In the current study, we observed a reduction in the expression and signaling of TGF-β1 and an increase in the expression of IL-1β mRNA in the mPFC of NVHL animals during neonatal and post-pubertal periods suggesting a persistent increase of inflammatory load in distant connected structures following neonatal lesion of the VH. Notably, the lesion did not produce inflammatory reaction in other structures not directly connected, e.g., SSC nor in the brain tissues surrounding the lesion site. This suggests a particular vulnerability of the hippocampal-PFC circuit following the lesion possibly due to a loss of the ventral hippocampal excitatory drive to the PFC. Our data provide a mechanistic explanation to the growing evidence suggesting an important role of the developing VH inputs in shaping adult PFC physiology and behavior (Liu and Carter, 2018).

The neonatal VH lesioned rats have been previously shown to display a number of cellular and behavioral changes linked to the PFC (Swerdlow et al., 2001; Chambers and Taylor, 2004). In our recent study, we provided the evidence of increased microglial reactivity, phagocytic activity and inflammation in the PFC of NVHL rats (Hui et al. under review). Microglia are a major source of early inflammatory genes like IL-1β within the CNS and IL-1β imbalance has been associated with cognitive and other behavioral deficits (Murray et al., 2013; Tsai, 2017). We believe that the increased expression of IL-1β mRNA might be a key factor in regulating the behavioral and synaptic development in lesioned animals during adulthood. We acknowledge that not measuring IL-1β protein levels is a limitation in our study as it could have further strengthened our conclusions; however, taken together, our data suggest a key role of IL-1β in contributing to the inflammatory overload observed within the PFC. Upregulation of canonical TGF-β signaling pathway (via p-Smad-2/3) was found to contribute to quiescent microglia phenotype thereby reducing inflammatory overload within CNS (Abutbul et al., 2012; Kierdorf and Prinz, 2013). IL-1β exerts a suppressive effect on TGF-β1 mediated Smad-2/3 phosphorylation (Benus et al., 2005; Roman-Blas et al., 2007; Lim et al., 2012), suggesting that the reduction of TGF-β1 in PFC of lesion animals observed in our study may be due to the suppressive effect of elevated IL-1β mRNA on TGF-β signaling. Thus, we hypothesized that neonatal administration of TGF-β1 in NVHL animals could rescue the inflammation-driven schizophrenia- related behavioral and cellular deficits in NVHL animals.

Pro-inflammatory cytokines are tightly regulated by anti-inflammatory cytokines; IL-1β must overcome the anti-inflammatory effects of TGF-β1 to boost pro-inflammatory responses (Coussens and Werb, 2002). Our data shows that neonatal TGF-β1 administration attenuated the increase in IL-1β mRNA in the PFC of lesioned animals. TGF-β1 has been reported to inhibit IL-1β signaling by disrupting the Pellino1/IRAK1 complex by Smad6 (Choi et al., 2006). Thus, suggesting that neonatal administration of recombinant TGF-β1 could be playing a critical role in counterbalancing the imbalance of IL-1β-TGF-β1 within PFC in lesion animals. NVHL-induced hyperlocomotion, deficits in PPI and SIs in the post-pubertal animals were also rescued by the administration of TGF-β1at neonatal age. Moreover, neonatal TGF-β1 also prevented the loss of spines observed in the mPFC of lesion animals. Reduced dendritic spine density in the PFC pyramidal neurons has been shown in both human schizophrenia brains as well as in the NVHL animals (Glantz and Lewis, 2000; Ryan et al., 2013) and suggested to alter glutamatergic and GABAergic neurotransmission and cognitive dysfunctions (Lewis and González-Burgos, 2008; Selemon, 2013). *In vitro* and *in vivo* studies provide evidence towards the role of TGF-β1 regulating synaptogenesis in cortical and thalamic neurons (Diniz et al., 2012; Bialas and Stevens, 2013). TGF-β signaling pathway plays a critical role in neuron specification and its disruption has been identified as one of the factors contributing to neurodevelopmental disorders such as schizophrenia (Yi et al., 2010; Nakashima et al., 2018). Apart from its role in synaptic growth, TGF-β signaling has been suggested to maintain synaptic homeostasis. Sun’s group used a Smad-4 knockout mouse model to show that TGF-β signaling deficits leads to an imbalance in the synaptic homeostasis in the hippocampus resulting in behavioral abnormalities such as hyperactivity and deficits in PPI (Sun et al., 2010). Further, microglia has been reported to work synergistically along with TGF-β1 to regulate microglia mediated synaptic pruning (Bialas and Stevens, 2013).Thus, it is possible that reduced TGF-β1 could be a key player in the synaptic abnormalities observed in the PFC of NVHL animals.

Although our data shows a functional effect of systemically administered recombinant TGF-β1, similar to what Caraci et al. (2015) observed, it should be pointed out that Kastin’s group has reported that circulating TGF-β1 does not cross the blood brain barrier (BBB) in adult rats (Kastin et al., 2003). However, they also showed that TGF-β1 can cross BBB when experiments were done in serum free condition, suggesting that TGF-β1 is brain- penetrant under certain conditions, at least in the adult animals. Thus, in our experiments it was important to demonstrate that the recombinant TGF-β1 reaches the brain in the neonatal period and the behavioral results that we obtained are indeed due to the effect of recombinant TGF-β1 in the brain. We observed increased phosphorylation of Smad-2/3, a key intracellular signaling partner of TGF-β1, in the TGF-β1 injected animals as compared to the saline injected animals (Figure 5). Accordingly, we believe that due to immaturity of the BBB in neonates, TGF-β1 likely crosses the BBB and exerts its signaling effects resulting in the behavioral rescue that we observed. It should be pointed out that we do not know the actual concentration of injected TGFβ1 in the brain; however, the activation of Smad-2/3 does suggest a physiological meaningful action of the injected recombinant TGF-β1.

Our data are consistent with recent evidence pointing towards an important role of neuroinflammation in neurodevelopmental disorders such as schizophrenia (Sekar et al., 2016; Fan and Pang, 2017). A meta-analysis of cytokines in the CSF showed similar results, i.e., higher levels of pro- and lower levels of anti-inflammatory cytokines in patients with schizophrenia (Wang and Miller, 2018). Evidence supporting the role of increased inflammation towards NVHL adult behavioral deficits has been provided in a previous study (Drouin-Ouellet et al., 2011). Interestingly, in our study the inflammatory overload is only observed during early development (around PD15) within the mPFC (a region distant from the lesion site) which is a critical time point of circuit development. In summary, our study shows that TGF-β1 may act as a neuroprotective agent to confer protection against the NVHL-induced behavioral, cellular and molecular deficits and suggests a closer examination of anti-inflammatory growth factors in the pathology of disorders such as schizophrenia.

## Author Contributions

AJ conceived, designed and performed the experiments and also wrote the first draft of the manuscript. SB helped in performing experiments and data analysis and manuscript writing. LS contributed to the development of research idea and implementation, discussion of the results and part of the manuscript writing.

## Conflict of Interest Statement

The authors declare that the research was conducted in the absence of any commercial or financial relationships that could be construed as a potential conflict of interest.

## References

[B1] Abutbul S. Shapiro J. Szaingurten-Solodkin I. Levy N. Carmy Y. Baron R.. (2012). TGF-β signaling through SMAD2/3 induces the quiescent microglial phenotype within the CNS environment. Glia 60, 1160–1171. 10.1002/glia.22343 22511296

[B2] Adzic M. Brkic Z. Mitic M. Francija E. Jovicic M. J. Radulovic J.. (2018). Therapeutic strategies for treatment of inflammation-related depression. Curr. Neuropharmacol. 16, 176–209. 10.2174/1570159x15666170828163048 28847294 PMC5883379

[B3] Andreou K. E. Soto M. S. Allen D. Economopoulos V. de Bernardi A. Larkin J. R.. (2017). Anti-inflammatory microglia/macrophages as a potential therapeutic target in brain metastasis. Front. Oncol. 7:251. 10.3389/fonc.2017.00251 29164051 PMC5670100

[B4] Baharnoori M. Brake W. G. Srivastava L. K. (2009). Prenatal immune challenge induces developmental changes in the morphology of pyramidal neurons of the prefrontal cortex and hippocampus in rats. Schizophr. Res. 107, 99–109. 10.1016/j.schres.2008.10.003 19004618

[B5] Bähner F. Meyer-Lindenberg A. (2017). Hippocampal-prefrontal connectivity as a translational phenotype for schizophrenia. Eur. Neuropsychopharmacol. 27, 93–106. 10.1016/j.euroneuro.2016.12.007 28089652

[B6] Basu A. Krady J. K. Enterline J. R. Levison S. W. (2002). Transforming growth factor β1 prevents IL-1β-induced microglial activation, whereas TNFα- and IL-6-stimulated activation are not antagonized. Glia 40, 109–120. 10.1002/glia.10118 12237848

[B7] Behrens M. M. Ali S. S. Dao D. N. Lucero J. Shekhtman G. Quick K. L.. (2007). Ketamine-induced loss of phenotype of fast-spiking interneurons is mediated by NADPH-oxidase. Science 318, 1645–1647. 10.1126/science.1148045 18063801

[B8] Benus G. F. Wierenga A. T. de Gorter D. J. Schuringa J. J. van Bennekum A. M. Drenth-Diephuis L.. (2005). Inhibition of the transforming growth factor β (TGFβ) pathway by interleukin-1β is mediated through TGFβ-activated kinase 1 phosphorylation of SMAD3. Mol. Biol. Cell 16, 3501–3510. 10.1091/mbc.e04-11-1033 15917296 PMC1182292

[B9] Bhardwaj S. K. Forcelli P. A. Palchik G. Gale K. Srivastava L. K. Kondratyev A. (2012). Neonatal exposure to phenobarbital potentiates schizophrenia-like behavioral outcomes in the rat. Neuropharmacology 62, 2337–2345. 10.1016/j.neuropharm.2012.02.001 22366076 PMC3318973

[B10] Bhardwaj S. K. Tse Y. C. Ryan R. Wong T. P. Srivastava L. K. (2014). Impaired adrenergic-mediated plasticity of prefrontal cortical glutamate synapses in rats with developmental disruption of the ventral hippocampus. Neuropsychopharmacology 39, 2963–2973. 10.1038/npp.2014.142 24917197 PMC4229566

[B11] Bialas A. R. Stevens B. (2013). TGF-β signaling regulates neuronal C1q expression and developmental synaptic refinement. Nat. Neurosci. 16, 1773–1782. 10.1038/nn.3560 24162655 PMC3973738

[B12] Bitzer-Quintero O. K. González-Burgos I. (2012). Immune system in the brain: a modulatory role on dendritic spine morphophysiology? Neural Plast. 2012:348642. 10.1155/2012/348642 22548192 PMC3324176

[B13] Brockmann M. D. Pöschel B. Cichon N. Hanganu-Opatz I. L. (2011). Coupled oscillations mediate directed interactions between prefrontal cortex and hippocampus of the neonatal rat. Neuron 71, 332–347. 10.1016/j.neuron.2011.05.041 21791291

[B14] Butovsky O. Jedrychowski M. P. Moore C. S. Cialic R. Lanser A. J. Gabriely G.. (2014). Identification of a unique TGF-β-dependent molecular and functional signature in microglia. Nat. Neurosci. 17, 131–143. 10.1038/nn.3599 24316888 PMC4066672

[B15] Cabungcal J.-H. Counotte D. S. Lewis E. M. Tejeda H. A. Piantadosi P. Pollock C.. (2014). Juvenile antioxidant treatment prevents adult deficits in a developmental model of schizophrenia. Neuron 83, 1073–1084. 10.1016/j.neuron.2014.07.028 25132466 PMC4418441

[B16] Caraci F. Gulisano W. Guida C. A. Impellizzeri A. A. Drago F. Puzzo D.. (2015). A key role for TGF-β1 in hippocampal synaptic plasticity and memory. Sci. Rep. 5:11252. 10.1038/srep11252 26059637 PMC4462026

[B17] Chambers R. A. Taylor J. R. (2004). Animal modeling dual diagnosis schizophrenia: sensitization to cocaine in rats with neonatal ventral hippocampal lesions. Biol. Psychiatry 56, 308–316. 10.1016/j.biopsych.2004.05.019 15336512

[B18] Choi K.-C. Lee Y. S. Lim S. Choi H. K. Lee C.-H. Lee E.-K.. (2006). Smad6 negatively regulates interleukin 1-receptor-Toll-like receptor signaling through direct interaction with the adaptor Pellino-1. Nat. Immunol. 7, 1057–1065. 10.1038/ni1383 16951688

[B19] Coussens L. M. Werb Z. (2002). Inflammation and cancer. Nature 420, 860–867. 10.1038/nature01322 12490959 PMC2803035

[B20] Deverman B. E. Patterson P. H. (2009). Cytokines and CNS development. Neuron 64, 61–78. 10.1016/j.neuron.2009.09.002 19840550

[B21] Diniz L. P. Almeida J. C. Tortelli V. Vargas Lopes C. Setti-Perdigão P. Stipursky J.. (2012). Astrocyte-induced synaptogenesis is mediated by transforming growth factor β signaling through modulation of D-serine levels in cerebral cortex neurons. J. Biol. Chem. 287, 41432–41445. 10.1074/jbc.M112.380824 23055518 PMC3510841

[B22] Dobolyi A. Vincze C. Pál G. Lovas G. (2012). The neuroprotective functions of transforming growth factor β proteins. Int. J. Mol. Sci. 13, 8219–8258. 10.3390/ijms13078219 22942700 PMC3430231

[B23] Drouin-Ouellet J. Brownell A. L. Saint-Pierre M. Fasano C. Emond V. Trudeau L. E.. (2011). Neuroinflammation is associated with changes in glial mGluR5 expression and the development of neonatal excitotoxic lesions. Glia 59, 188–199. 10.1002/glia.21086 21125661 PMC3983848

[B24] Fan L.-W. Pang Y. (2017). Dysregulation of neurogenesis by neuroinflammation: key differences in neurodevelopmental and neurological disorders. Neural Regen. Res. 12, 366–371. 10.4103/1673-5374.202926 28469641 PMC5399704

[B25] Flores G. Alquicer G. Silva-Gómez A. B. Zaldivar G. Stewart J. Quirion R.. (2005). Alterations in dendritic morphology of prefrontal cortical and nucleus accumbens neurons in post-pubertal rats after neonatal excitotoxic lesions of the ventral hippocampus. Neuroscience 133, 463–470. 10.1016/j.neuroscience.2005.02.021 15878241

[B26] Flores G. Barbeau D. Quirion R. Srivastava L. K. (1996). Decreased binding of dopamine D3 receptors in limbic subregions after neonatal bilateral lesion of rat hippocampus. J. Neurosci. 16, 2020–2026. 10.1523/JNEUROSCI.16-06-02020.1996 8604046 PMC6578499

[B27] Gilmore J. H. Fredrik Jarskog L. Vadlamudi S. Lauder J. M. (2004). Prenatal infection and risk for schizophrenia: IL-1β, IL-6, and TNFα inhibit cortical neuron dendrite development. Neuropsychopharmacology 29, 1221–1229. 10.1038/sj.npp.1300446 15085088

[B28] Glantz L. A. Lewis D. A. (2000). Decreased dendritic spine density on prefrontal cortical pyramidal neurons in schizophrenia. Arch. Gen. Psychiatry 57, 65–73. 10.1001/archpsyc.57.1.65 10632234

[B29] Godsil B. P. Kiss J. P. Spedding M. Jay T. M. (2013). The hippocampal-prefrontal pathway: the weak link in psychiatric disorders? Eur. Neuropsychopharmacol. 23, 1165–1181. 10.1016/j.euroneuro.2012.10.018 23332457

[B30] Goverman J. (2009). Autoimmune T cell responses in the central nervous system. Nat. Rev. Immunol. 9, 393–407. 10.1038/nri2550 19444307 PMC2813731

[B31] Jia P. Wang L. Meltzer H. Y. Zhao Z. (2010). Common variants conferring risk of schizophrenia: a pathway analysis of GWAS data. Schizophr. Res. 122, 38–42. 10.1016/j.schres.2010.07.001 20659789 PMC2933424

[B32] Kastin A. J. Akerstrom V. Pan W. (2003). Circulating TGF-β1 does not cross the intact blood-brain barrier. J. Mol. Neurosci. 21, 43–48. 10.1385/jmn:21:1:43 14500993

[B33] Kierdorf K. Prinz M. (2013). Factors regulating microglia activation. Front. Cell. Neurosci. 7:44. 10.3389/fncel.2013.00044 23630462 PMC3632747

[B34] Kondo S. Kohsaka S. Okabe S. (2011). Long-term changes of spine dynamics and microglia after transient peripheral immune response triggered by LPS *in vivo*. Mol. Brain 4:27. 10.1186/1756-6606-4-27 21682853 PMC3138393

[B35] Lewis D. A. González-Burgos G. (2008). Neuroplasticity of neocortical circuits in schizophrenia. Neuropsychopharmacology 33, 141–165. 10.1038/sj.npp.1301563 17805309

[B36] Lim S. Bae E. Kim H.-S. Kim T.-A. Byun K. Kim B.. (2012). TRAF6 mediates IL-1β/LPS-induced suppression of TGF-β signaling through its interaction with the type III TGF-β receptor. PLoS One 7:e32705. 10.1371/journal.pone.0032705 22427868 PMC3299683

[B37] Liu X. Carter A. G. (2018). Ventral hippocampal inputs preferentially drive cortico-cortical neurons in the infralimbic prefrontal cortex. J. Neurosci. 38, 7351–7363. 10.1523/JNEUROSCI.0378-18.2018 29959235 PMC6096040

[B38] Marcotte E. R. Pearson D. M. Srivastava L. K. (2001). Animal models of schizophrenia: a critical review. J. Psychiatry Neurosci. 26, 395–410. 11762207 PMC167198

[B39] McKibben C. E. Reynolds G. P. Jenkins T. A. (2014). Analysis of sociability and preference for social novelty in the acute and subchronic phencyclidine rat. J. Psychopharmacol. 28, 955–963. 10.1177/0269881114544778 25122039

[B40] Murray C. L. Obiang P. Bannerman D. Cunningham C. (2013). Endogenous IL-1 in cognitive function and anxiety: a study in IL-1RI^−/−^ mice. PLoS One 8:e78385. 10.1371/journal.pone.0078385 24205219 PMC3813582

[B41] Nakashima M. Imada H. Shiraishi E. Ito Y. Suzuki N. Miyamoto M.. (2018). Phosphodiesterase 2A inhibitor TAK-915 ameliorates cognitive impairments and social withdrawal in N-methyl-d-aspartate receptor antagonist-induced rat models of schizophrenia. J. Pharmacol. Exp. Ther. 365, 179–188. 10.1124/jpet.117.245506 29440309

[B42] Roman-Blas J. A. Stokes D. G. Jimenez S. A. (2007). Modulation of TGF-β signaling by proinflammatory cytokines in articular chondrocytes. Osteoarthritis Cartilage 15, 1367–1377. 10.1016/j.joca.2007.04.011 17604656 PMC2153443

[B43] Ryan R. T. Bhardwaj S. K. Tse Y. C. Srivastava L. K. Wong T. P. (2013). Opposing alterations in excitation and inhibition of layer 5 medial prefrontal cortex pyramidal neurons following neonatal ventral hippocampal lesion. Cereb. Cortex 23, 1198–1207. 10.1093/cercor/bhs111 22581849

[B44] Sekar A. Bialas A. R. de Rivera H. Davis A. Hammond T. R. Kamitaki N.. (2016). Schizophrenia risk from complex variation of complement component 4. Nature 530, 177–183. 10.1038/nature16549 26814963 PMC4752392

[B45] Selemon L. D. (2013). A role for synaptic plasticity in the adolescent development of executive function. Transl. Psychiatry 3:e238. 10.1038/tp.2013.7 23462989 PMC3625918

[B46] Spittau B. Krieglstein K. (2012). Klf10 and Klf11 as mediators of TGF-β superfamily signaling. Cell Tissue Res. 347, 65–72. 10.1007/s00441-011-1186-6 21574058

[B47] Spulber S. Bartfai T. Schultzberg M. (2009). IL-1/IL-1ra balance in the brain revisited - evidence from transgenic mouse models. Brain Behav. Immun. 23, 573–579. 10.1016/j.bbi.2009.02.015 19258032

[B48] Sun M. Gewirtz J. C. Bofenkamp L. Wickham R. J. Ge H. O’Connor M. B. (2010). Canonical TGF-β signaling is required for the balance of excitatory/inhibitory transmission within the hippocampus and prepulse inhibition of acoustic startle. J. Neurosci. 30, 6025–6035. 10.1523/JNEUROSCI.0789-10.2010 20427661 PMC6632596

[B49] Swerdlow N. R. Geyer M. A. Braff D. L. (2001). Neural circuit regulation of prepulse inhibition of startle in the rat: current knowledge and future challenges. Psychopharmacology 156, 194–215. 10.1007/s002130100799 11549223

[B50] Thierry A. M. Gioanni Y. Degenetais E. Glowinski J. (2000). Hippocampo-prefrontal cortex pathway: anatomical and electrophysiological characteristics. Hippocampus 10, 411–419. 10.1002/1098-1063(2000)10:4<411::aid-hipo7>3.0.co;2-a 10985280

[B51] Trépanier M. O. Hopperton K. E. Mizrahi R. Mechawar N. Bazinet R. P. (2016). Postmortem evidence of cerebral inflammation in schizophrenia: a systematic review. Mol. Psychiatry 21, 1009–1026. 10.1038/mp.2016.90 27271499 PMC4960446

[B52] Tsai S. J. (2017). Effects of interleukin-1β polymorphisms on brain function and behavior in healthy and psychiatric disease conditions. Cytokine Growth Factor Rev. 37, 89–97. 10.1016/j.cytogfr.2017.06.001 28599834

[B53] Tseng K. Y. Chambers R. A. Lipska B. K. (2009). The neonatal ventral hippocampal lesion as a heuristic neurodevelopmental model of schizophrenia. Behav. Brain Res. 204, 295–305. 10.1016/j.bbr.2008.11.039 19100784 PMC2735579

[B54] Wang A. K. Miller B. J. (2018). Meta-analysis of cerebrospinal fluid cytokine and tryptophan catabolite alterations in psychiatric patients: comparisons between schizophrenia, bipolar disorder, and depression. Schizophr. Bull. 44, 75–83. 10.1093/schbul/sbx035 28338954 PMC5768046

[B55] Wang W.-Y. Tan M.-S. Yu J.-T. Tan L. (2015). Role of pro-inflammatory cytokines released from microglia in Alzheimer’s disease. Ann. Transl. Med. 3:136. 10.3978/j.issn.2305-5839.2015.03.49 26207229 PMC4486922

[B56] Wei H. Chadman K. K. McCloskey D. P. Sheikh A. M. Malik M. Brown W. T.. (2012). Brain IL-6 elevation causes neuronal circuitry imbalances and mediates autism-like behaviors. Biochim. Biophys. Acta 1822, 831–842. 10.1016/j.bbadis.2012.01.011 22326556

[B57] Yamato M. Tamura Y. Eguchi A. Kume S. Miyashige Y. Nakano M.. (2014). Brain interleukin-1β and the intrinsic receptor antagonist control peripheral Toll-like receptor 3-mediated suppression of spontaneous activity in rats. PLoS One 9:e90950. 10.1371/journal.pone.0090950 24621600 PMC3951245

[B58] Yi J. J. Barnes A. P. Hand R. Polleux F. Ehlers M. D. (2010). TGF-β signaling specifies axons during brain development. Cell 142, 144–157. 10.1016/j.cell.2010.06.010 20603020 PMC2933408

